# PRENATAL MUSCLE FORCES ARE NECESSARY FOR VERTEBRAL SEGMENTATION AND DISC STRUCTURE, BUT NOT FOR NOTOCHORD INVOLUTION IN MICE

**DOI:** 10.22203/eCM.v041a36

**Published:** 2021-05-22

**Authors:** A. Levillain, S. Ahmed, D-M. Kaimaki, S. Schuler, S. Barros, D. Labonte, J.C. Iatridis, N.C. Nowlan

**Affiliations:** 1 Department of Bioengineering, Imperial College London, London, UK; 2 Université de Lyon, Université Claude Bernard Lyon 1, INSERM, LYOS UMR 1033, Lyon, France; 3 Department of Orthopaedics, Icahn School of Medicine at Mount Sinai, New York, NY, USA; 4 School of Mechanical and Materials Engineering, University College Dublin, Dublin, Ireland; 5 UCD Conway Institute, University College Dublin, Dublin, Ireland

**Keywords:** Intervertebral disc – development, spine – biomechanics, notochord, muscular dysgenesis, embryo, paralysis, ECM – collagens

## Abstract

Embryonic muscle forces are necessary for normal vertebral development and spinal curvature, but their involvement in intervertebral disc (IVD) development remains unclear. The aim of the current study was to determine how muscle contractions affect (1) notochord involution and vertebral segmentation, and (2) IVD development including the mechanical properties and morphology, as well as collagen fibre alignment in the annulus fibrosus. Muscular dysgenesis (mdg) mice were harvested at three prenatal stages: at Theiler Stage (TS)22 when notochord involution starts, at TS24 when involution is complete, and at TS27 when the IVD is formed. Vertebral and IVD development were characterised using histology, immunofluorescence, and indentation testing. The results revealed that notochord involution and vertebral segmentation occurred independently of muscle contractions between TS22 and TS24. However, in the absence of muscle contractions, we found vertebral fusion in the cervical region at TS27, along with (i) a displacement of the nucleus pulposus towards the dorsal side, (ii) a disruption of the structural arrangement of collagen in the annulus fibrosus, and (iii) an increase in viscous behaviour of the annulus fibrosus. These findings emphasise the important role of mechanical forces during IVD development, and demonstrate a critical role of muscle loading during development to enable proper annulus fibrosus formation. They further suggest a need for mechanical loading in the creation of fibre-reinforced tissue engineering replacement IVDs as a therapy for IVD degeneration.

## Introduction

Intervertebral discs (IVDs), due to their unique structure, play an important role in the biomechanics of the spine, as they carry loads, dissipate energy and facilitate joint mobility (Smith *et al.*, 2011). IVDs are comprised of a core gelatinous nucleus pulposus (NP) surrounded by a lamellar ring-like annulus fibrosus (AF). Both elements act synergistically to distribute and transmit loads between vertebral bodies. Degenerated IVDs display a decrease in their proteoglycan content, an increase in the percentage of denatured type II collagen, and a disorganised extracellular matrix (Antoniou *et al.*, 1996). These changes alter the AF mechanical properties, in turn impairing the mechanical function of the IVD (Emanuel *et al.*, 2018). IVD degeneration mainly affects cervical and lumbar regions, and is associated with neck and low back pain (reviewed in Kushchayev *et al.*, 2018). In the absence of treatments to restore disc structure and mechanical functions, tissue engineering of replacement discs is a promising strategy. In the past few years, research has focused on deriving cartilage and bone tissue from undifferentiated cells by using biochemical and mechanical cues from development (Gadjanski *et al.*, 2012); a process coined “developmental engineering” (Alkhatib *et al.*, 2018), and in the IVD, factors from development can promote regeneration and prevent painful conditions (Purmessur *et al.*, 2013). Application of this approach to the IVD is hampered by complexity of its structure and a lack of understanding of the mechanisms driving IVD development. Several studies have focused on the roles of specific genes and transcription factors in IVD development (reviewed in Alkhatib *et al.*, 2018), as well as the contribution of extracellular matrix components, such as collagen XII and XIV and small leucine-rich proteoglycan, to tissue architecture and biomechanical properties of the intervertebral discs (Caldeira *et al.*, 2017; Rajasekaran *et al.*, 2021). By contrast, few studies have investigated the role of mechanical forces in driving IVD development (Aszódi *et al.*, 1998). The importance of mechanical stimuli for tissue engineering other biological load-bearing tissues such as cartilage is well-established (reviewed in Li *et al.*, 2017 and Salinas *et al.*, 2018). While mechanical overloading may promote maturation of notochordal cells (Guehring *et al.*, 2010), detailed knowledge on the role of mechanical stimuli in IVD development is limited, but may inform and lead to advances in IVD tissue engineering.

Several lines of evidence indicate a role for mechanical factors during embryonic development of the NP and AF, which are formed concurrently but follow distinct developmental pathways (Smith *et al.*, 2011). The NP, whose cells can be distinguished from AF and articular cartilage cells by their high expression of N-cadherin (Lv *et al.*, 2013), is derived from the notochord, which contracts within the forming vertebral bodies and expands towards the future disc – a process called “involution” (Smith *et al.*, 2011). Mechanical forces coming from the expansion of the amniotic cavity are important for the convergent extension of the notochord during morphogenesis (Imuta *et al.*, 2014) and are hypothesised to also play a role in notochord involution (Williams *et al.*, 2019). Indeed, in the absence of collagen II, which restrains the swelling pressure in the vertebral bodies and enables the cartilage to resist compressive forces, vertebral bodies of embryonic mice fail to form normally and the notochord retains its original continuous rod-like morphology (Aszódi *et al.*, 1998). Similarly, when the notochord sheath is absent following removal of Sonic Hedgehog (*Shh*) in mice, notochord cells fail to migrate towards the future NP and become scattered within the vertebral bodies (Choi *et al.*, 2008; Choi and Harfe, 2011). Based on these observations, a “pressure model” has been proposed (Choi and Harfe, 2011), suggesting that the internal swelling pressure of the forming vertebral bodies induces notochordal compression, and the notochord sheath constrains the notochord cells along the vertebral column. There is also some evidence to suggest that the lamellar structure of the AF may be, at least in part, mechanically determined. The AF, along with the vertebral bodies, is derived from the sclerotome, which adopts a metameric pattern of more and less condensed regions (Scaal, 2016). A study comparing spine development in chick, (where the notochord does not undergo involution), and mouse embryos proposed that collagen fibre alignment in the AF derives from the bulging pattern of the notochord (Ghazanfari *et al.*, 2018). It has been proposed that further mechanical signals due to onset of muscle activity in the spine lead to reinforcement of the lamellar structure of the AF (Ghazanfari *et al.*, 2018; Hayes *et al.*, 2011). This hypothesis is supported by studies on patients with adolescent idiopathic scoliosis (AIS), in which abnormal spinal and peripheral muscles are associated with a disorganization of the lamellar structure of the AF (reviewed in Wise *et al.*, 2020). However, despite these hypotheses, the role and the origin of these biomechanical forces during IVD development remain unclear.

A key source of mechanical stimuli during vertebral development are embryonic muscle contractions. Previous work by the authors describes how prolonged or short-term paralysis of chick embryos results in abnormal spinal curvature, wedged and fused vertebral bodies, as well as rib defects (Levillain *et al.*, 2019; Rolfe *et al.*, 2017). However, the chick model did not allow the study of IVD development, as notochord involution does not occur in this model (Shapiro, 1992). A small number of studies have been conducted on spine development in the murine Pax3 mutant Splotch (Henderson *et al.*, 1999; Schubert *et al.*, 2001; Tremblay *et al.*, 1998), characterised by absence of limb musculature and further abnormalities in hypaxial muscles (Tremblay *et al.*, 1998). Splotch mouse embryos display fused vertebral bodies and IVDs with reduced height at embryonic day (E) 18.5 (Schubert *et al.*, 2001). These studies suggest a role of muscle forces for spine and disc development, but since Pax3 mutations also result in neural crest deficiencies and neural tube defects (Tremblay *et al.*, 1998), it is difficult to conclude to what extent abnormal muscle forces are responsible for the observed spinal defects. The muscular dysgenesis (mdg) mouse, which carries a naturally occurring mutation in the *CACNA1S* gene, is characterised by a lack of excitation-contraction coupling, leading to an absence of skeletal muscle contraction and resulting in paralysis (Pai, 1965). Mdg embryos have vertebral fusion dorsally in cervical and lumbar regions at E18.5 (Kahn *et al.*, 2009) and fused vertebral bodies in the cervical and thoracic regions at birth (Pai, 1965). These changes are likely associated with IVD defects, but the relationships between vertebral body and IVD abnormalities remain unknown. Furthermore, neither the role of muscle forces in notochord involution, nor the effects of abnormal muscle forces on the morphology, collagen alignment and mechanical properties of the IVD have been investigated, but all are critical for IVD functionality.

The aim of this study was to determine how the absence of muscle contractions affects disc development in mice. Based on the findings on abnormal vertebral shape and segmentation in the chick (Levillain *et al.*, 2019; Rolfe *et al.*, 2017), it was first hypothesised that muscle contractions during murine prenatal development are needed for a) normal vertebral segmentation and (b) normal vertebral shape and mechanical properties. Hence, abnormalities in either or both aspects of spine development are expected to cause abnormal notochord involution. Second, in line with the evidence suggesting a role of biomechanical forces for NP and AF development, it was further hypothesised that muscle activity is necessary for normal development of the IVD, including disc morphology and mechanical properties, as well as alignment of collagen fibres in the AF. The first hypothesis was tested by comparing vertebral segmentation and notochord involution in mdg and control mice at the developmental stages of initiation and completion of involution [TS22 and TS24 (Smith *et al.*, 2011)], as well as mechanical properties of the vertebral bodies at TS24. The second hypothesis was tested by comparing the morphological and mechanical properties of mdg and control IVDs at TS27, when the IVD is fully formed.

## Materials and Methods

### Tissue collection and processing

**Fig. 1.**
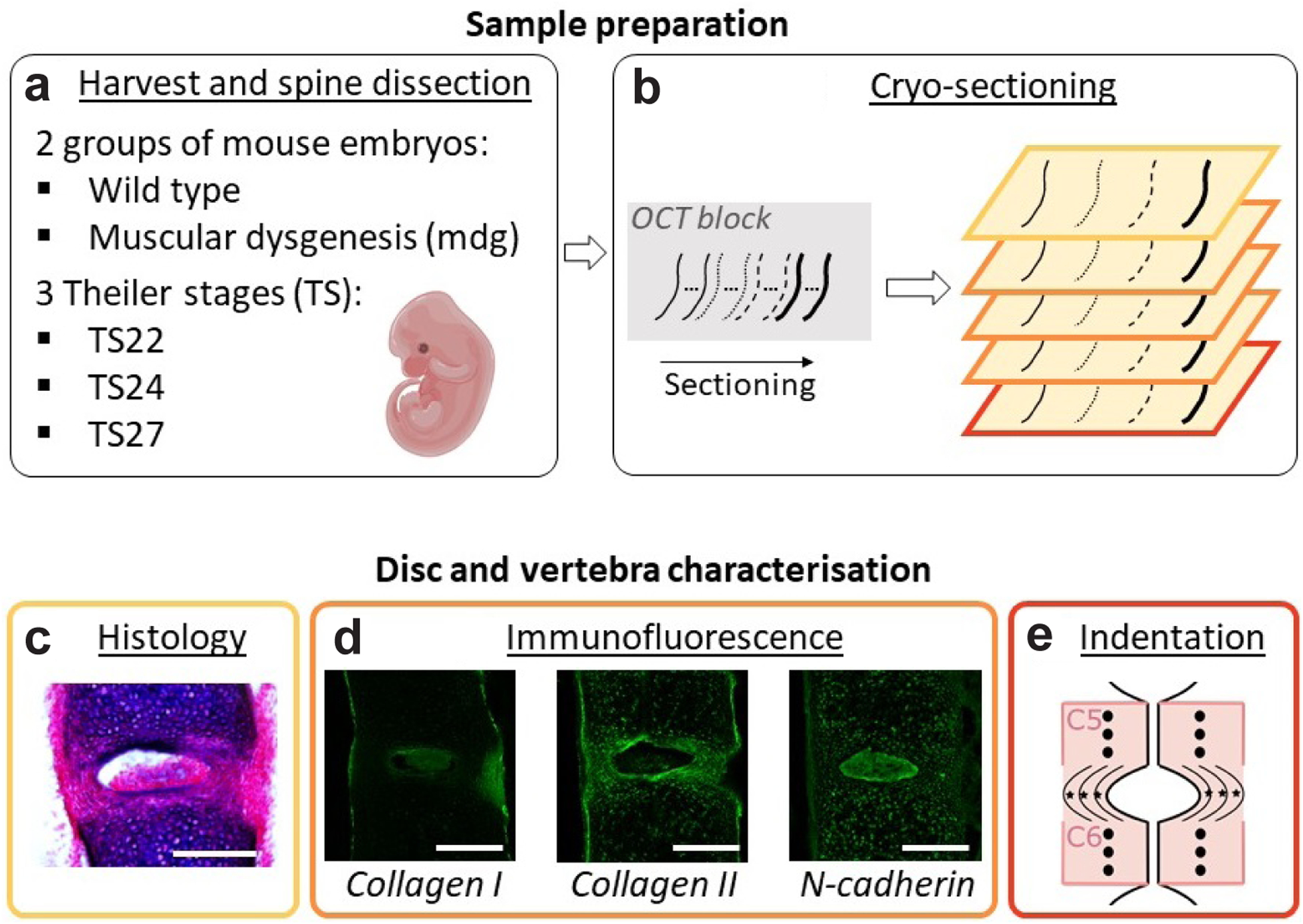
Methods overview. (**a**) Wild type and muscular dysgenesis (mdg) mouse embryos were harvested at Theiler stages (TS) 22, TS24, and TS27. The spine was carefully dissected. (**b**) 25 μm-thick frozen serial sections were cut and collected on 5 consecutive slides, starting with sections represented by the thin plain line, following by sections represented by the dotted line, dashed line, and thick plain line. In this way, the 5 slides were almost identical. (**c**) Sections from 1 slide (yellow in **b**) were stained with alcian blue and picrosirius red to assess vertebral segmentation in the cervical, thoracic, and lumbar regions. (**d**) Immunofluorescence analyses were performed on sections from 3 slides (orange in **b**), using collagen I, collagen II, and N-cadherin antibodies. Morphology of each component of the disc was characterised in the cervical region. (**e**) Mechanical properties of the vertebrae and AF were assessed on 1 section from the last slide (red in **b**) using instrumented indentation. Approximate locations of indents in the vertebral body (black circle) and AF (black star) of intervertebral discs (sagittal view) are illustrated. Indentation was performed on vertebral bodies (6 to 10 indents per vertebra) and AF located between the cervical C5 and C6 (6 indents per AF). Scale bar = 200 μm.

All experiments were performed in accordance with European legislation (Directive 2010/63/EU). The mdg (Pai, 1965) line was kindly obtained from E. Zelzer (Department of Molecular Genetics, Weizmann Institute of Science, Israel) and was bred to C57Bl/6J mice. Heterozygous females and males were mated to provide litters containing mdg homozygous embryos, from now on referred to as ‘mdg embryos’. Wild type C57BL/6 embryos (both littermates and fully wild-type litters) were used as controls. All embryos were harvested and staged according to Theiler Stages TS22, TS24 and TS27 (typically embryonic day 13.5, 15.5 and 18.5, respectively) (Fig. 1a). Genotyping was done by PCR on DNA derived from ear (adult mice) or head (embryos) tissue. The PCR reaction was carried out for 30 cycles, each with a duration of 1 min at 94 °C, 65 °C and 72°C, using the primer pair 5’-CCGAGCTGAGGAGACACTTG and 3’- GGGCATATGTGGTACCAGCA (ThermoFisher) and the diluted PCR product (1 : 10) was sequenced (University of Dundee, Dundee, UK) using the forward primer. Spines were carefully dissected, cryo-protected in increasing sucrose gradients (15 % and 30 %) until the samples sank, and then embedded in a 50 : 50 mix containing 30 % sucrose and optimal cutting temperature (OCT) compound (Agar Scientific, Stansted, UK). 25 μm-thick frozen serial sections were cut with a cryostat (NX70, Leica Biosystems) and collected on five consecutive slides for histology (one slide), immunofluorescence (three slides), and indentation testing (one slide) (Fig. 1b). A minimum of three mice per developmental stage were analysed for both control and mdg groups (Table 1).

### Histology

Sections used for histology were fixed in 4 % paraformaldehyde (PFA) (VWR, Lutterworth, UK) for 20 min, stained with 0.025 % alcian blue (Merck) in 3 % acetic acid (Merck) (for cartilage) for 30 min followed by 1 % picrosirius red (Merck) (for collagen) for 30 min (Fig. 1c) (Rolfe *et al.*, 2017). Sections from the cervical, thoracic, and lumbar regions were imaged in transmitted illumination using a light microscope (Yenway EX30, Glasgow, UK), with light intensity being adjusted to observe the vertebral bodies. Vertebral segmentation, disc morphology, and vertebral shape were qualitatively compared between control and mdg spines, using light microscopy images.

### Immunofluorescence and image acquisition

Sections used for immunofluorescence were permeabilised with 0.1 % Tween-20 (Merck)/1 % dimethyl sulphoxide (Merck) in phosphate buffered saline (PBS), blocked with 5 % normal goat serum (Merck) and incubated with a primary antibody (1 : 100 dilution) overnight at 4 °C (Ahmed and Nowlan, 2020). Based on studies looking at the immunohistochemical localisation of collagens in embryonic mouse spine (Aszódi *et al.*, 1998) and specific molecular markers for the NP (Lv *et al.*, 2013), the following primary antibodies were used (at all stages unless specified): rabbit anti-collagen I (ab34710, Abcam) for the outer AF at TS24 and TS27 (Aszódi *et al.*, 1998), mouse anti-collagen II (MAB8887, Merck) for the vertebral bodies and inner AF (Aszódi *et al.*, 1998), rabbit anti-collagen III (ab7778, Abcam) for the notochord sheath at TS22 (Aszódi *et al.*, 1998), and rabbit anti-N-cadherin (ab18203, Abcam) for the notochord and NP (Lv *et al.*, 2013). Sections were then washed and incubated with secondary antibody (1 : 400 dilution) for 2 h and counterstained with DAPI (1 : 2,000 dilution) for 30 s. The following secondary antibodies were used: goat anti-mouse Alexa Fluor^®^ 488 (A–11029, Thermo Fisher Scientific), and goat anti-rabbit Alexa Fluor^®^ 488 (A–11008, Thermo Fisher Scientific). Direct fluorescence acquisition of labelled tissue sections was performed using a Leica SP8 Inverted Confocal Laser Scanning Microscope (Leica Microsystems) equipped with a Helium-Neon 633 nm laser. The gain and offset were adjusted manually to collect the optimum fluorescence data and prevent saturation. Stacks of images of the cervical region (C5–C6) were acquired using a × 20 objective (HC PL APO 20×/0.75 CS2) and reconstructed in Fiji to produce maximum intensity Z-projections (Fig. 1d).

### Image analysis

Z-projection images of collagen II obtained through confocal microscopy were saved as TIF files and used for further analysis to compare the organisation of the inner AF between three control and three mdg embryos at TS24 and TS27 (when the IVD is formed). The inner AF C5–C6 was manually cropped in ImageJ (NIH, Bethesda, Maryland, USA). The resulting image was then divided into four equal regions: cranial dorsal, cranial ventral, caudal dorsal, and caudal ventral. In each region, the orientation of collagen fibrils was quantified using the OrientationJ plugin in ImageJ (Rezakhaniha *et al.*, 2012). The local orientation (relative to the dorsoventral axis) of each pixel with an intensity larger than 5 % of the maximum intensity and a coherency larger than 0.05 was calculated using the cubic spline gradient method, with a Gaussian window of 10 pixels (Rezakhaniha *et al.*, 2012). For each stage and each region, collagen orientation distribution was plotted in circular histograms using Matlab (MathWorks^®^, R2015a, Natick, MA, USA) and compared between control and mdg embryos. Due to the small sample size (*n* = 3), no statistical analysis was performed.

### Indentation testing

**Fig. 2.**
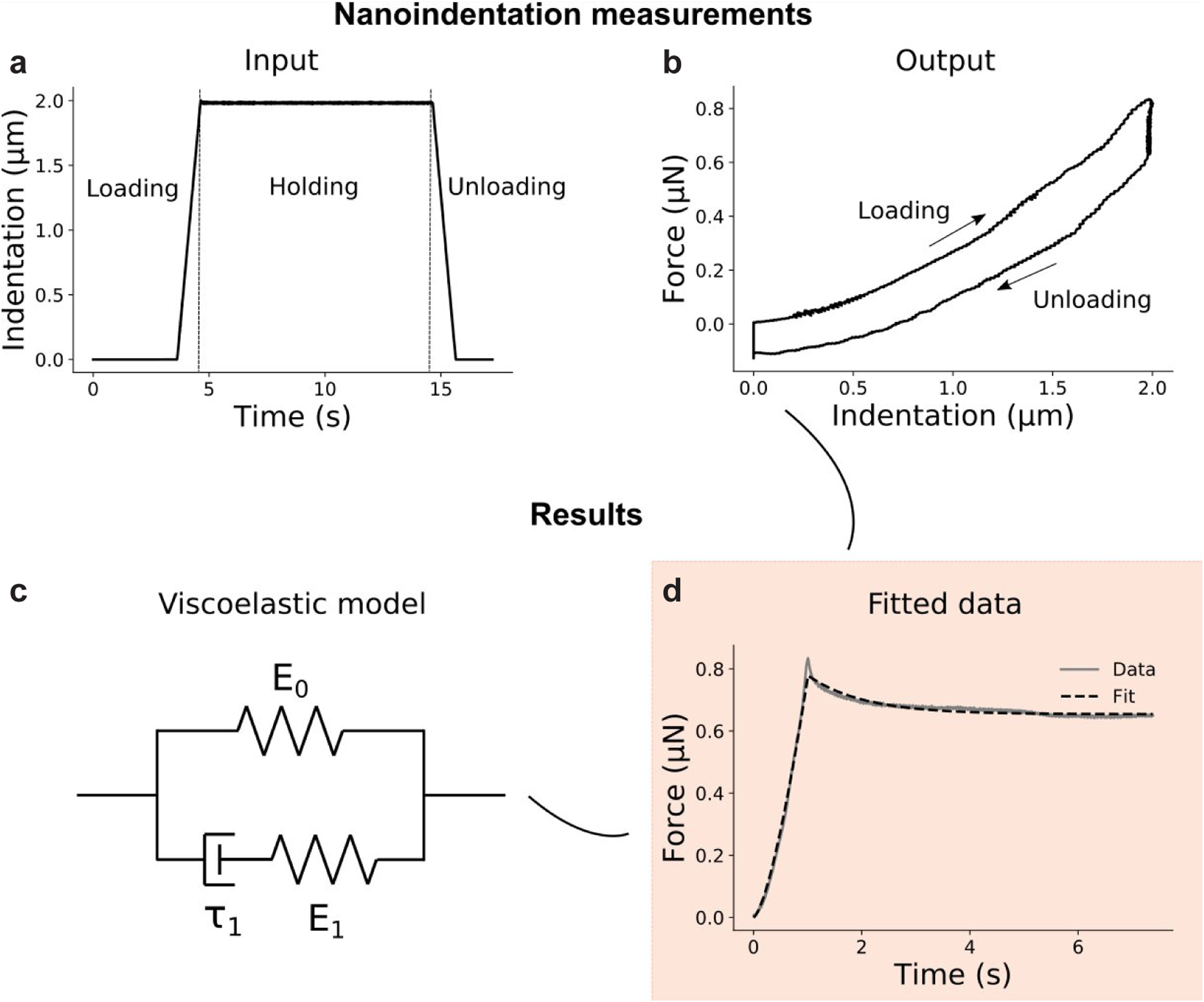
Indentation method. (**a**) Indentation profile. In the loading phase, the displacement was increased at a constant rate of 2 μm/s until it reached 2 μm. The displacement was then held for 10 s and unloading was carried out at 2 μm/s. (**b**) Output force-indentation curve showing the loading and unloading phases. (**c**) Viscoelastic model. The material was represented as a spring in parallel to a Maxwell solid (a spring and dashpot connected in series). (**d**) The relaxation function was determined by fitting the resultant experimental force-time curve on the loading and holding segments. E_0_ , E_1_: elastic moduli associated with each spring; τ_1_: time constant associated with the dashpot.

Sections used for instrumented indentation were thawed at room temperature for 30 min before testing. To characterise the time-dependent behaviour of the tissue, indentation tests in closed-loop displacement-control were performed on sections immersed in PBS using a Chiaro nanoindentor (Optics 11, Amsterdam, the Netherlands). A 0.48 N/m probe with a spherical tip of 42 μm radius was chosen on the basis of the expected material properties and dimensions of the samples (Web ref. 1). A trapezoidal indentation profile was used (Fig. 2a), comprised of a loading phase at 2 μm/s until the displacement reached a maximum value of 2 μm. The maximum displacement was chosen to avoid excessive strains (less than 5 % of indentation strain) and substrate effects (indentation depth less than 10 % of the sample thickness) (Qiang *et al.*, 2011). The displacement was then held constant for 10 s and unloading was carried out at 2 μm/s (Fig. 2a). Tests were conducted on the same region as for immunofluorescence (*i.e*. vertebral bodies C5 and C6 and the inner AF C5–C6) from a mid-sagittal section of three control and mdg embryos at TS24 and TS27 (when the IVD is formed) (Fig. 1e). To account for spatial heterogeneity in the material properties, a minimum of six indents was performed for each structure (vertebral bodies and AF), with a minimal spacing of 100 μm between each indent to prevent interactions from neighbouring indents. For each sample, the duration of indentation testing did not exceed 1 h to minimise potential glycosaminoglycan leakage (Perie *et al.*, 2006).

### Viscoelastic analysis

To investigate the time-dependence of the mechanical response, elastic-viscoelastic correspondence was used, following previous work (Lee and Radok, 1960; Mattice *et al.*, 2006; Oyen, 2006; Sakai, 2002). In brief, the elastic operator in the Hertzian contact model was replaced with a Boltzman hereditary integral operator, which includes a relaxation function, for which a Maxwell standard linear solid was chosen (Fig. 2c). The hereditary integral was then solved separately for ramp- and hold-phase (Oyen, 2006), and the solution was fitted to the experimental data (Fig. 2d) to extract (i) the instantaneous modulus, *E*_*ins*′_ (ii) the equilibrium modulus, *E*_*eq*′_ [assuming incompressibility (Marturano *et al.*, 2013)], and (iii) the elastic fraction, *f* = *E*_*eq*_*/E*_*ins*′_ which quantifies the extent of viscous behaviour of the material (an elastic fraction of 0 corresponds to a purely viscous material, while an elastic fraction of 1 corresponds to a purely elastic material) (Mattice *et al.*, 2006; Oyen, 2006). A contact criterion needs to be defined for indentation of a soft tissue (Marturano *et al.*, 2013). Here, contact with the sample was considered when a significant change in the slope of the force indentation curve occurred (0.003 μN or about 0.5 % of maximum force) (Fig. 2b).

Statistical analyses were performed in R 3.6.2 (R foundation for Statistical Computing, Vienna, Austria). Statistical comparisons between the viscoelastic properties (instantaneous modulus and the elastic fraction) of age-matched control and mdg groups were made on the total number of indents using a linear mixed model with a level of significance of 0.05. Group (Control/mdg) was considered as a fixed factor while sample ID was considered as a random factor. Values that deviated from the sample mean by more than three standard deviations were excluded from the analysis.

## Results

**Fig. 3.**
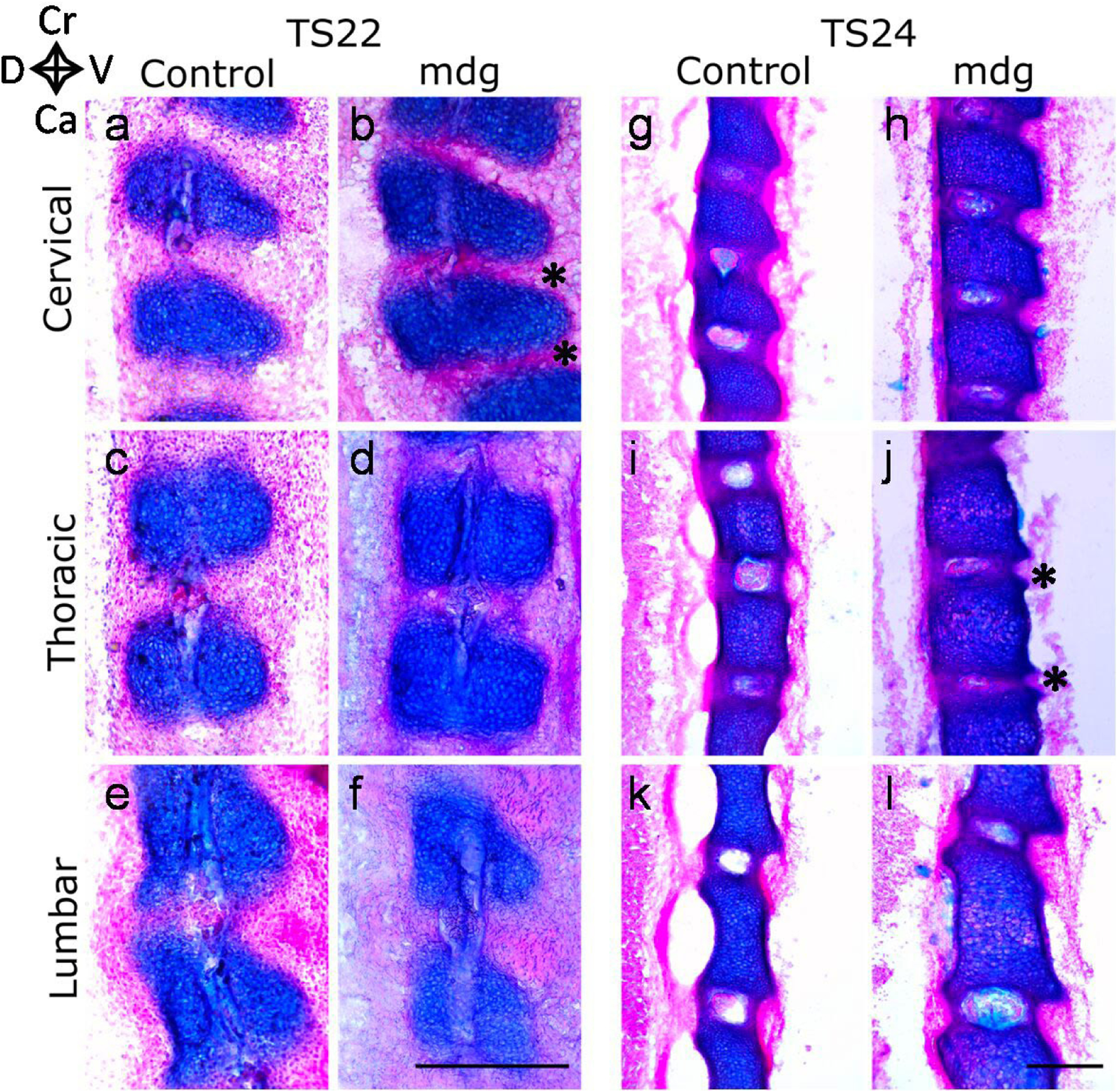
The lack of muscle contractions did not affect vertebral segmentation or notochord involution at TS22 or TS24. Notochord involution initiated at TS22 (**a**–**f**) and was complete at TS24 in mdg (**h**,**j**,**l**) and control (**g**,**i**,**k**) samples. Vertebral bodies (stained in blue) were clearly separated by the AF in all regions. In mdg samples, reduced intervertebral spaces (*) were observed in the cervical region at TS22 (**b**) and in the thoracic region at TS24 (**j**) compared to the controls (**a** and **i**, respectively). (**a**–**f**) 25 μm-thick sagittal spine sections stained with alcian blue (cartilage) and picrosirius red (collagen) of representative control and mdg samples at TS22 and (**g**–**l**) at TS24, in the cervical, thoracic, and lumbar regions. Scale bars: 500 μm. Ca: caudal; Cr: cranial; D: dorsal; V: ventral.

Results were reasonably consistent between mdg embryos at TS22 and TS24, and therefore representative images for one specimen are shown for these stages (Fig. 3), with any variation between samples reported in the text. At TS27, there was a high degree of variability between mdg samples and therefore data from all TS27 control and mdg embryos are shown. Moreover, results were consistent across consecutive sections, so only one section per specimen is shown.

### Vertebral segmentation and disc formation

Initiation of notochord involution at TS22 was equivalent in mdg and control samples (Fig. 3a–f), and was most progressed in the cervical region (Fig. 3a–b). Vertebral bodies were clearly separated from the developing AF for both mdg and control embryos (Fig. 3), demonstrating that vertebral segmentation occurred in all regions despite the absence of muscle contractions. However, the intervertebral space appeared reduced in the mdg embryos compared to the control samples (Fig. 3b* *vs*. Fig. 3a), especially in the cervical region.

At TS24, notochord involution completed normally in all examined regions of mdg embryos (Fig. 3g–l). The NP was formed and surrounded by the forming AF, divided into the inner AF composed of glycosaminoglycan and collagen, and the collagen-rich outer AF (Fig. 3g–l). The mdg embryos still displayed a reduced intervertebral space on the ventral side of the thoracic region compared to control samples (Fig. 3j* *vs*. Fig. 3i), while vertebral spacing was similar between control and mdg embryos in the cervical and lumbar regions (Fig. 3g–h,k–l).

**Fig. 4.**
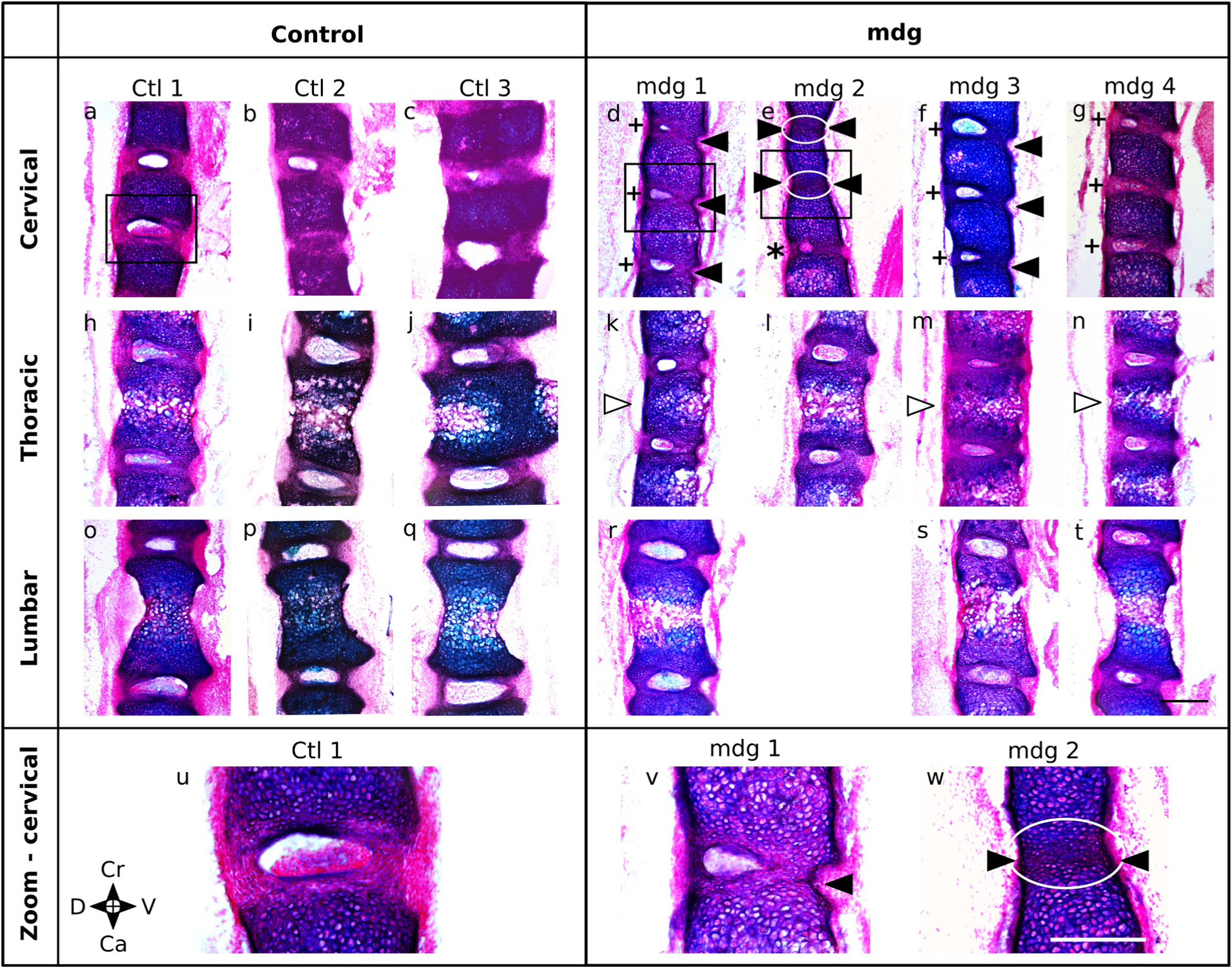
Absence of muscle contraction resulted in morphological defects of the intervertebral discs at most spinal levels with partial or complete vertebral fusion in the cervical region at TS27, with subtle changes in the thoracic region, and no major changes were in the lumbar region. Due to variability in the mdg group, all 4 mdg samples are shown and compared with all 3 control samples. In the cervical region, there was vertebral fusion (black arrowhead) in *3* out of 4 mdg samples, to varying degree of severity, from complete vertebral fusion in 1 sample (**e**,**w**) to partial vertebral fusion on the ventral side in 2 samples (**d**,**f**,**v**). In 1 particularly severe case, absence of contractile muscles resulted in a disruption of NP formation (**e**,**w**), with no NP between C4 and C6 (white ellipse) and a reduced NP between C6 and C7 (*). In the 3 other mdg samples (**d**,**f**,**g**), the NP was decentred towards the dorsal side (**+**). In the thoracic region, 3 mdg samples displayed wedged vertebral bodies (white arrowhead), while no changes were observed in the lumbar region. (**a**-**t**): 25 μm-thick sagittal spine sections stained with alcian blue (cartilage) and picrosirius red (collagen) at TS27, in the cervical (C5–C7 or C4–C7), thoracic (T3–T5), and lumbar (L2–L4) regions of control and mdg spines. No suitable section was obtained for the lumbar region of ‘mdg 2’ due to a staining issue. (**u**–**w**): Zoomed-in specific regions (black boxes) of (**a**–**t**). *S*cale bars = 500 μm. Ca: caudal; Cr: cranial; D: dorsal; V: ventral.

At TS27, vertebral fusion and abnormal positioning of the NP in the cervical region were observed in the mdg embryos, while other regions were mildly affected by the absence of muscle contractions (Fig. 4). One mdg embryo (‘mdg 2’) showed complete vertebral fusion between C4 and C6 (Fig. 4e,w – black arrowhead), with no NP between these vertebrae (Fig. 4e,w – white box). The disc was absent only in this sample and in these locations. mdg 2 also had a NP with reduced size compared to control samples between cervical C6 and C7 (Fig. 4e – *), while the NPs located between C2 and C4 appeared normal (data not shown). Two out of the three remaining mdg embryos (‘mdg 1’ and ‘mdg 3’) displayed partial vertebral fusion on the ventral sides of the discs (Fig. 4d,f,v – black arrowhead), while vertebral bodies of all control samples were clearly separated on the ventral and dorsal sides by the AF (Fig. 4a–c). Moreover, where present, the NPs of all mdg embryos were off-centred towards the dorsal side (Fig. 4d,f,g – **+**), while they were centred along the dorsoventral axis in control samples (Fig. 4a–c). In the thoracic region, the NPs appeared smaller than controls and sometimes off-centred (Fig. 4k,m *vs.* 4h–c,j); most of the vertebral bodies of the mdg embryos appeared wedged (Fig. 4k,m,n – white arrowhead). In the lumbar region (Fig. 4r–t), no fusion was observed in any of the mdg embryos, and the vertebral bodies and NPs did not show obvious deformities.

### AF and NP formation and morphology in cervical region

**Fig. 5.**
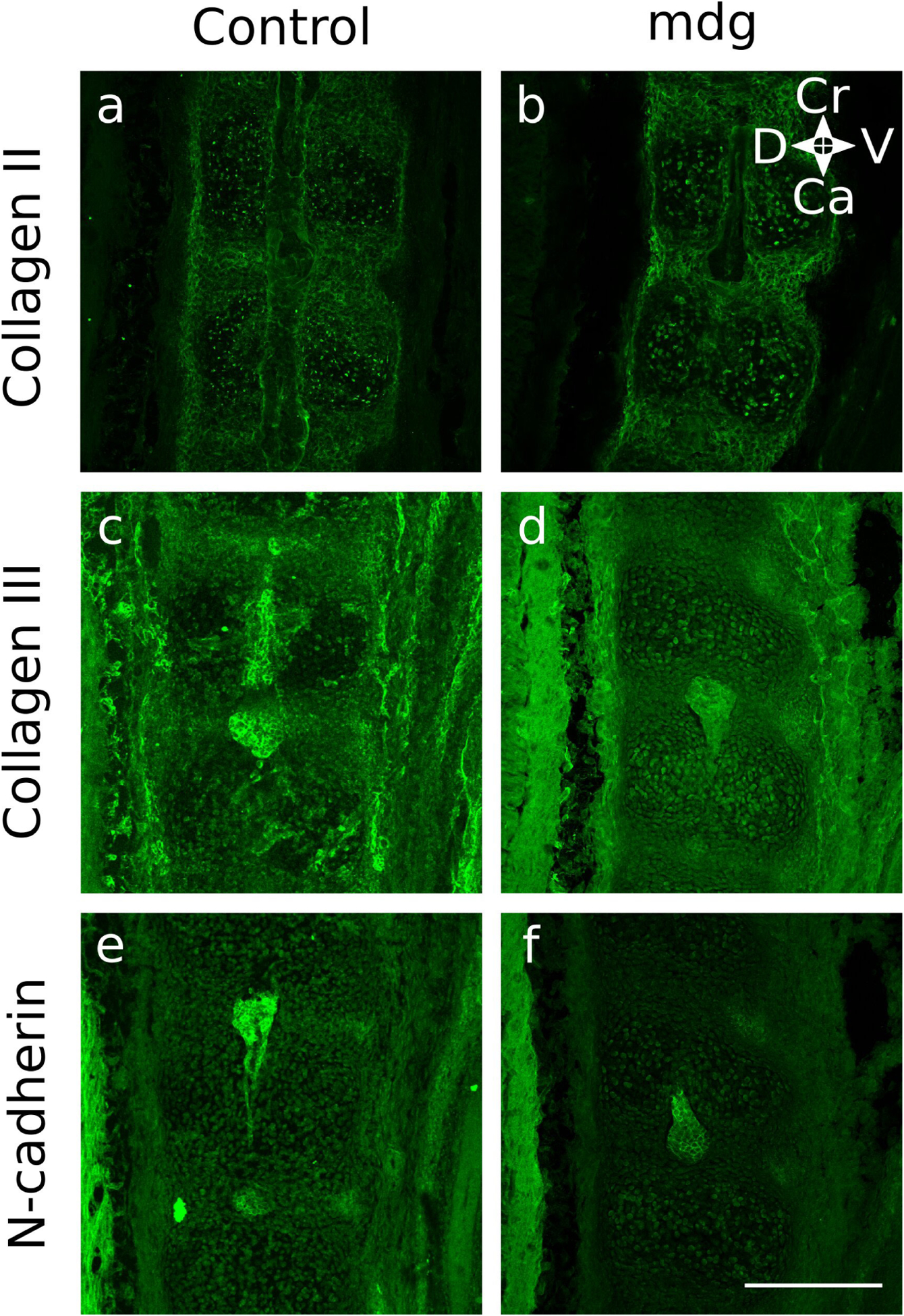
Formation of notochord sheath and initiation of notochord involution at TS22 were not dependent on muscle contractions. The notochord formed normally in the mdg group, and was enveloped in a continuous type II collagen-rich notochord sheath that extended along the craniocaudal axis **(b).** Notochord cells expressed N-cadherin and started migrating towards the development site of NP (**f**). Cervical region of representative TS22 control and mdg spines shown (sagittal view). Due to the size of the samples and the small numbers of sections showing the notochord, different segments of the cervical region were imaged. Scale bar = 200 μm. Ca: caudal; Cr: cranial; D: dorsal; V: ventral.

**Fig. 6.**
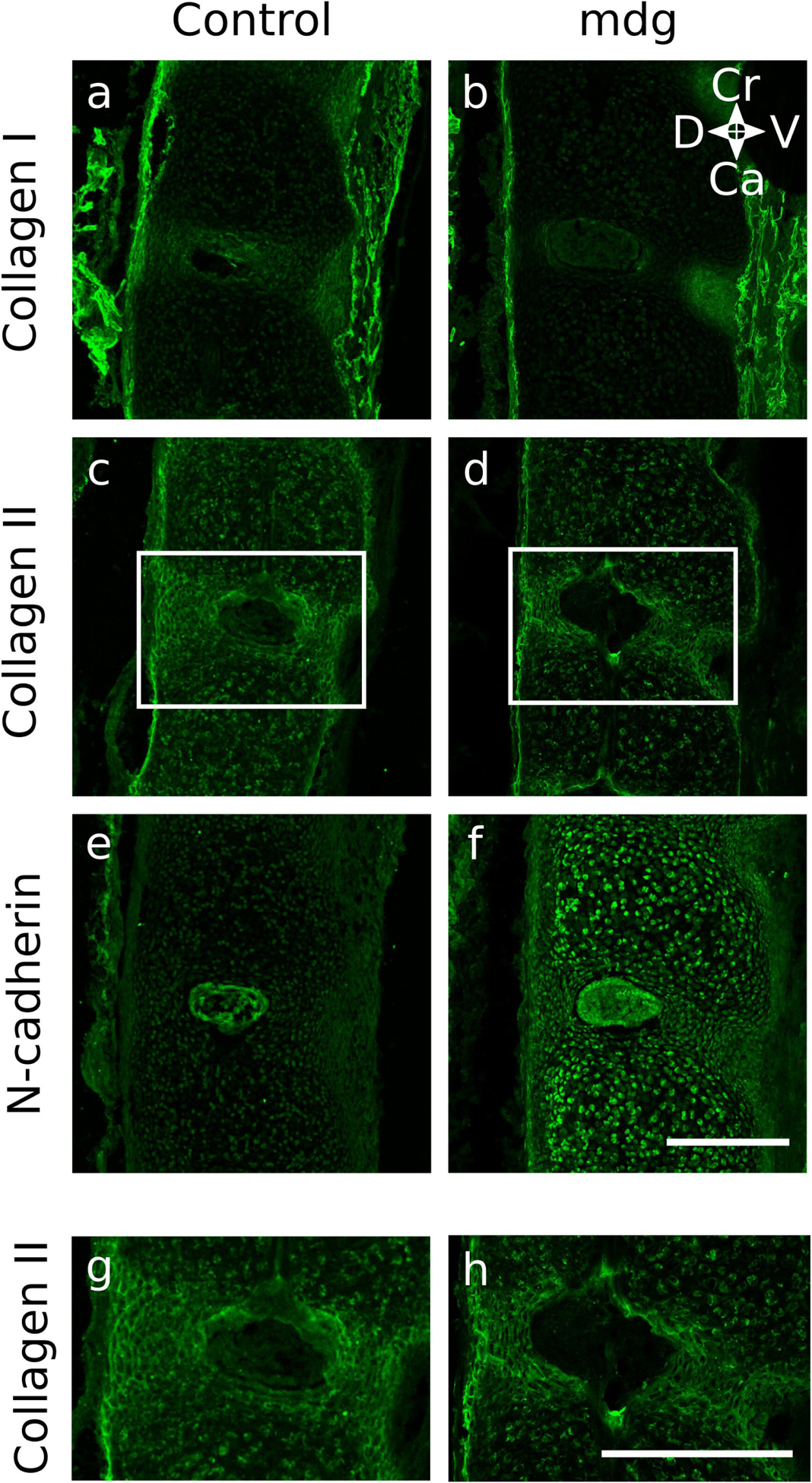
Muscle activity was not required for formation of each component of the intervertebral disc at TS24, but ectopic expression of N-cadherin was observed in 2 out of 3 mdg samples. The NP formed normally in mdg samples (**f**), indicating completion of notochord involution, and the AF was divided into a collagen II-rich inner part showing a lamellar arrangement (**d**,**h**) and a collagen I-rich outer part (**f**). *W*hile N-cadherin was expressed only in the NP of control spines, it was scattered throughout the spine in 2 out of 3 mdg samples (**f**). (**a**–**f**) Cervical region (C5–C6) of representative control and mdg spines shown (sagittal view). (**g**–**h**) Zoomed in view of collagen II expression showing the lamellar structure in the inner AF (regions shown from white boxes in **c**–**d**). *S*cale bars = 200 μm. Ca: caudal; Cr: cranial; D: dorsal; V: ventral.

**Fig. 7.**
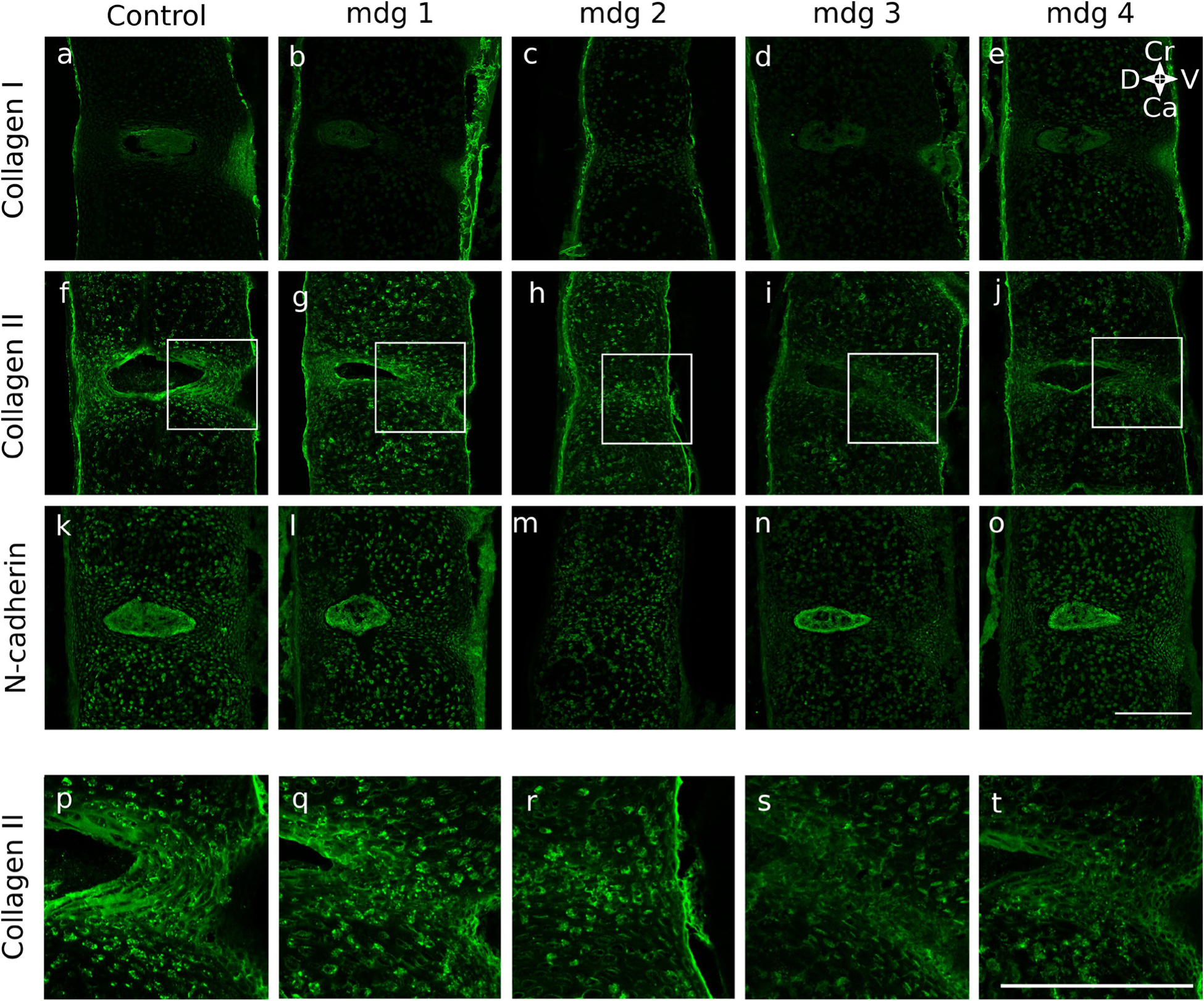
Muscle activity was required for the lamellar arrangement of the inner AF at TS27, and some discs were highly abnormal in the mdg samples. All mdg samples displayed dense collagen II signal in the inner AF (**g**–**j**), but the lamellar structure was affected to varying degrees. One mdg sample showed a complete lack of arrangement between cervical C3 and C6 (**h**,**r**), while others displayed a partial lack of arrangement on the ventral side (**g**,**i**,**q**,**s**) or a less prominent structure (**j**,**t**) compared to the control (**f**,**p**). The outer AF and NP formed normally in mdg specimens and expressed collagen I and N-cadherin, respectively, except in 1 severe case in which both structures were absent between cervical C3 and C6 (**c**,**m**). (**a**–**o**) Collagen I (**a**–**e**), collagen II (**f**–**j**), and N-cadherin (**k**–**o**) in the cervical region (C5–C6) of TS27 control (**a**,**f**,**k**) and mdg spines **b**–**e**, **g**–**j**, **l**–**o**). Due to variability in the mdg group, all 4 mdg samples are shown and compared with 1 representative control. (**p**–**t**) Zoomed in view of collagen II expression in ventral outer AF illustrating loss of lamellar structure in mdg specimens (regions shown from white boxes in **f**–**j**). Ca: caudal; Cr: cranial; D: dorsal; V: ventral. Scale bars = 200 μm.

When NP formation was assessed using immunofluorescent markers, it was found that morphology was dependent on muscle contractions in only some samples. At TS22, the notochord in all mdg embryos expressed N-cadherin as normal (Fig. 5f), and was wrapped in a continuous type II collagen-rich notochord sheath extending along its craniocaudal axis (Fig. 5b). At TS24, the NP in mdg embryos continued to express N-cadherin (Fig. 6f), but in two out of three samples, expression of N-cadherin was scattered throughout the spine, instead of being limited to the NP (Fig. 6f). At TS27, N-cadherin signal was undetectable in one out of four mdg embryos in a single location (Fig. 7m), indicating absence of the NP, while other samples exhibited normal expression of N-cadherin in the NP (Fig. 7l,n,o).

The emerging lamellar structure of the inner AF appeared normal in all mdg embryos at TS22 and TS24, but was abnormal at TS27. At TS22, alternating areas of future vertebral body and future AF were observed along the craniocaudal axis, with stronger type II collagen immunopositivity in the future AF compared to the vertebral body (Fig. 5b). At TS24, the inner AF displayed a lamellar-like arrangement in all mdg embryos (Fig. 6d,h), with concentric layers made of collagen II, as in control samples (Fig. 6c,g). At TS27, all mdg embryos displayed stronger immunolocalisation of collagen II in the inner AF compared to the vertebral bodies (Fig. 7). However, the lamellar structure was dependent on muscle contraction in all samples, to varying degrees (Fig. 7p–t). In one particularly severe case, the lamellar arrangement was completely lacking between cervical C4 and C6 (Fig. 7h,r), while it was lacking only on the ventral side in two samples (Fig. 7g,i,q,s). The lamellar arrangement was present on both sides in the other mdg embryo (Fig. 7j,t), although less prominent compared to the controls (Fig. 7f,p).

The outer AF, visualised using collagen I labelling, displayed subtle changes due to the absence of muscle contractions. In all but one mdg embryo, the outer AF was wider on the ventral side at TS24 (Fig. 6b) and TS27 (Fig. 7b,d,e), as was also observed in the controls (Fig. 6a, 7a). However, the height of the ventral outer AF region appeared reduced compared to the control samples at both stages (Fig. 6a,b and 7a,b,d,e). At TS27, one mdg embryo did not show any collagen I signal between cervical C4 and C6, indicating the complete absence of outer AF (Fig. 7c), while the outer AF formed normally in other locations of the cervical region for that particular specimen (data not shown).

### Quantification of structural organisation of the inner AF

**Fig. 8.**
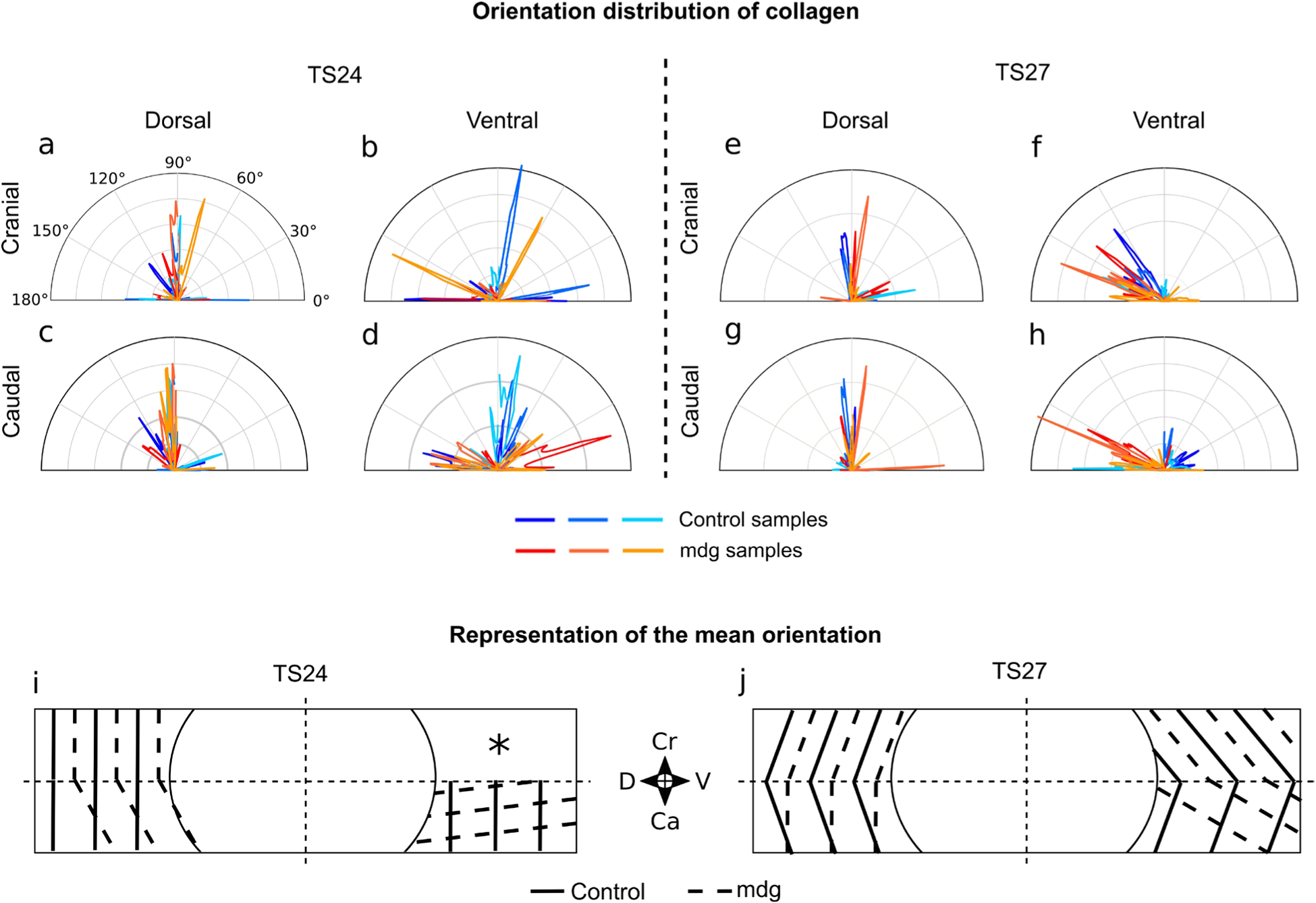
Collagen II orientation in the ventral side of the inner AF was disrupted in mdg samples, while its organisation was not affected on the dorsal side. On the ventral side of mdg samples, collagen II was orientated in a more oblique direction at TS24 compared to the controls, where it was mostly orientated in the vertical direction (**d**,**i**). At TS27, while collagen II in control samples displayed a circumferential pattern with opposite orientations in the cranial and caudal regions, collagen II in mdg samples had the same oblique orientation in both regions, indicating an absence of circumferential organisation (**f**,**h**,**j**). On the dorsal side, collagen II was mainly orientated in the cranio-caudal direction as in controls at both stages (**e**,**g**,**j**). (**a**–**h**) Circular histograms showing the distribution of collagen II orientation (relative to the dorsoventral axis) of 3 control and 3 mdg samples in 4 regions of the AF C5–C6 at TS24 and TS27. (**i**–**j**) Schematic of the AF showing the mean orientation of collagen II in the 4 regions examined of control (solid lines) and mdg (dashed lines) groups at TS24 and TS27 (*n* = 3 per group). *: no predominant orientation. Ca: caudal; Cr: cranial; D: dorsal; V: ventral.

A quantitative analysis of collagen II orientation corroborated our observation that the muscle contractions are required for collagen II orientation in the ventral, but not in the dorsal side of the inner AF (Fig. 8, Table 2). Fibre-orientations were affected in the caudal, but not in the cranial aspect. At TS24, in the cranial aspect, the orientation of collagen II fibres appeared similarly random in mdg and control groups (Fig. 8b,i). In the caudal aspect, collagen II fibres in the mdg group were predominantly orientated along the dorsoventral direction, with a mean orientation of 7 ± 13° relative to the dorsoventral axis, while in controls it was predominantly orientated in the craniocaudal direction, with a mean orientation of 87 ± 14° (Fig. 8d,i, Table 2). At TS27, collagen II orientation in the cranial aspect was again similar in the mdg and control groups (Fig. 8f), with mean orientations of 147 ± 7.8° and 132 ± 8.9°, respectively (Fig. 8j, Table 2); differences in orientation in the caudal aspect persisted. The mdg embryos showed a predominant orientation at 150 ± 4.9°, similar to the cranial aspect, indicating consistent collagen fibre orientation along the height of the disc (Fig. 8j, Table 2), while in the control group, the mean orientation of collagen II fibres in the control group was 53 ± 10° and opposite to the orientation in the cranial aspect, indicating a circumferential arrangement of collagen II on the ventral side (Fig. 8j, Table 2).

On the dorsal side of the AF, collagen II orientation was similar between groups throughout the height of the disc at both stages examined (Fig. 8a,c,e,g). Collagen II was mainly orientated in the craniocaudal direction, as evidenced by larger peaks at 90° (Fig. 8i,j).

### Vertebral body and AF mechanical properties

**Fig. 9.**
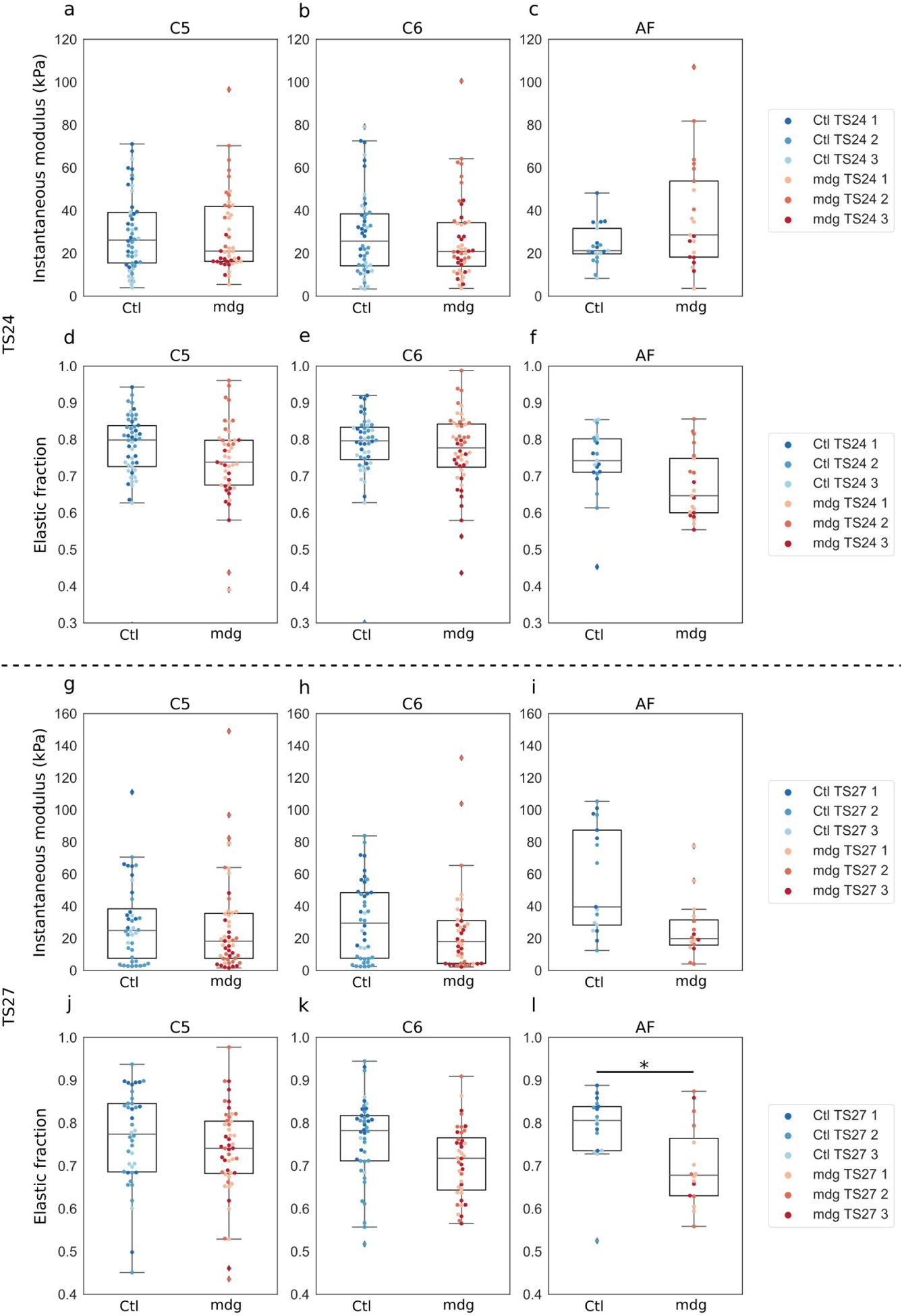
Elastic fraction of the AF at TS27 lower in mdg samples than in controls, while no effects on the vertebral bodies at TS24 and TS27, or on the AF at TS24, were observed **(a–c)**. Instantaneous modulus of the cervical vertebrae C5 and C6 and AF C5–C6 of control (blue) and mdg (red) embryos at TS24. (**d**–**f**) Elastic fraction of the cervical vertebrae C5 and C6 and AF C5–C6 of control (blue) and mdg (red) embryos at TS24. (**g**–**i**) Instantaneous modulus of the cervical vertebrae C5 and C6 and AF C5–C6 of control (blue) and mdg (red) embryos at TS27. (**j**–**l**) Elastic fraction of the cervical vertebrae C5 and C6 and AF C5–C6 of control (blue) and mdg (red) embryos at TS27. Dots represent individual data points (at least 6 indents per structure and per specimen) and colours represent specimens (*n* = 3 per group and per stage) * *p* < 0.05.

To evaluate a possible link between altered mechanical properties of the spinal unit tissues and altered disc and vertebral morphology in mdg mice, indentation tests were performed on the vertebral bodies C5 and C6 and the C5–C6 AF at TS24 and TS27 (Fig. 9, Table 3). The main difference found was in the TS27 AF, with a significantly reduced (*p* < 0.05) elastic fraction in the mdg group (Fig. 9l, Table 3), indicating an increase in viscoelasticity. There were no significant differences in instantaneous modulus or elastic fraction between mdg and control groups for either vertebral bodies or the AF at TS24 (Fig. 9a–f, Table 3). At TS27, there were still no significant differences in instantaneous modulus or elastic fraction for the vertebral bodies between groups (Fig. 9g–l, Table 3). Hence, the mechanical properties of the VB were not significantly affected by muscle contractions, suggesting that the pressure model is not related to muscle forces.

## Discussion

It was demonstrated that muscle contractions are necessary for late-, but not early-, stage disc development in mice. Therefore, the primary hypothesis, that prenatal muscle contractions are needed for early vertebral segmentation and morphogenesis events and that abnormalities in the vertebrae would lead to abnormal notochord involution, was not corroborated. Instead, vertebral bodies were normally segmented at TS22, and notochord involution occurred according to the normal timelines in all mdg embryos. The second hypothesis, that muscle activity is needed for normal development of the intervertebral disc, including morphology, alignment of collagen fibres in the AF, and mechanical properties, was corroborated. The inner AF of the TS27 disc had a disrupted lamellar arrangement and an increase in viscoelasticity in the cervical region, and the NP was displaced dorsally. Vertebral bodies were completely or partially fused in this region in most samples. These findings demonstrated that mechanical stimuli from muscle contractions are dispensable for disc specification and early disc development, but are required for later disc development events such as lamellar organisation.

The results of the present study offer new insights into the role of muscle activity for notochord involution and maintenance of the NP. Previous studies have emphasised the role of mechanical forces for the convergent extension and elongation of the notochord during morphogenesis and embryogenesis (Alkhatib *et al.*, 2018; Imuta *et al.*, 2014). Based on these findings, it has been proposed that mechanics-based mechanisms of development are also key for notochord involution (Rufai *et al.*, 1995). However, the origin of the biomechanical forces controlling involution remained unclear. It was demontrated that notochord involution took place between TS22 and TS24, both in absence and presence of muscle contractions, indicating that the biomechanical forces affecting notochord involution are intrinsic rather than extrinsic. Intrinsic compressive forces on the notochord may arise, for example, from the internal swelling pressure restrained by collagen fibrils in the vertebral bodies (Aszódi *et al.*, 1998). Indeed, notochord involution does not take place if collagen II is absent (Aszódi *et al.*, 1998), and the notochord sheath plays a key role in constraining notochord cells along the vertebral column (Choi and Harfe, 2011), consistent with this hypothesis (Choi and Harfe, 2011). It was shown that the notochord sheath formed normally in absence of muscle contraction at TS22 (when notochord involution begins) and that the mechanical properties of vertebral bodies did not differ significantly between mdg and control embryos at TS24 (when notochord involution is complete). Therefore, the findings suggest that the “pressure model” proposed in the literature is not related to muscle forces (Choi and Harfe, 2011). One mdg embryo was found where the NP was absent in two cervical locations (C4–C6) and highly reduced in another cervical location (C6–C7) at TS27. Since there was no evidence for N-cadherin signalling in the disc region or vertebral bodies in that sample, suggesting the absence of notochord cells, it is proposed that the NP disappeared rather than notochord involution occurring abnormally and notochord cells being scattered throughout the spine. One way that NP cells could disappear is hypertrophic differentiation. However, the NP cells did not seem to be morphologically different in the mdg mice compared to controls at initiation. Moreover, all mdg mice displayed stronger immunolocalisation of collagen II in the disc region compared to the vertebral bodies (Fig. 7q–t), and it has been shown that an increase in collagen X (which is expressed by hypertrophic cells) is associated with a decrease in collagen II (Lian *et al.*, 2019). Merceron *et al.* (2014) showed that in absence of the hypoxia inducible factor-1α (HIF-1α), the murine NP is formed at E15.5, but it progressively disappears postnatally following death of NP cells, and is replaced with fibrocartilage. Therefore, it is hypothesised that the NP disappeared after the death of NP cells. It is possible that in absence of muscle contraction, HIF-1*α* was highly downregulated in one of the mdg embryos, thus leading to the loss of the NP.

The results of this study highlight the importance of embryonic muscle forces for the structural arrangement of the AF. Although a lamellar arrangement of collagen II was initiated at TS24 in the inner AF, this pattern was partially or completely disrupted in the cervical region at TS27. A recent study comparing the AF structure in chick and mouse embryos suggested that mechanical stress induced by bulging of the notochord could be the mechanism underlying the criss-cross aligned pattern formation of collagen in the AF (Ghazanfari *et al.*, 2018). Hayes *et al.* (2011) proposed that the strengthening of the inner AF is derived from the onset of muscle activity in the spine, which causes compressive loading of the newly formed IVD. The current results support both hypotheses: in the absence of muscle contractions, collagen arrangement was initiated in the inner AF at TS24 following normal notochord involution, but it was disrupted at TS27. In contrast, the outer AF formed normally in most mdg embryos at TS27, which suggests that their development may be independent of mechanical stimuli provided by muscle contractions. Several studies demonstrated that Sox5/6 proteins and Pax1/9 signalling influences the division between inner and outer AF (Wise *et al.*, 2020). After division, the outer AF development is primarily regulated by Pax1 and Pax9 expressing cells, whereas the inner AF is primarily regulated by Sox9, which controls Sox5/6 (Wise *et al.*, 2020). An *in vitro* study on meniscus cells showed that Sox9 is downregulated in the absence of mechanical stretch (Kanazawa *et al.*, 2012). It is speculated that differential mechanoregulation of these genes may contribute to abnormal inner, but not outer, annulus structure when muscle forces are absent, but further studies are needed to test this hypothesis.

Changes in the structural arrangement of the AF were associated with altered viscoelastic properties. As far as is known, no data on the mechanical properties of the embryonic intervertebral disc are available in the literature. The instantaneous modulus of the disc, which quantifies the initial elastic response of the tissue, is consistent with the elastic modulus measured on other embryonic tissues, which ranges between 30 and 116 kPa for the femur of chick embryo between HH41 and HH43 (late stages of development) (Marturano *et al.*, 2013), and between 20 and 100 kPa for the developing murine growth plate cartilage between E13.5 and birth (Prein *et al.*, 2016). At TS27, both the instantaneous modulus and the elastic fraction of mdg embryos were reduced, while no difference was observed at TS24, suggesting a role of muscle forces for the emergence of mechanical properties of the disc. Pan *et al.* demonstrated that embryo movements regulate the mechanical properties of chick embryo tendons (Pan *et al.*, 2018), and that paralysis leads to a reduction in the modulus of the femur at a late stage of development, which supports the current findings. However, whether the change in the collagen structural arrangement of the AF and the alteration of the viscoelastic properties of the tissue happened chronologically or independently is unclear, as they were both normal at TS24 and abnormal at TS27. In the tendon of chick embryos, a change in collagen cross-linking results in a modification of the mechanical properties without affecting the collagen organisation (Marturano *et al.*, 2014). Moreover, adult degenerative discs display different collagen fibre organisation from healthy discs, but their tensile properties, which are primarily governed by collagen fibres, are similar (Vergari *et al.*, 2017). Based on these findings, it is proposed that the elastic fraction of the AF is independently affected by the absence of muscle contractions.

Remarkably, the findings of the current study suggest that the effect of non-contractile muscle on the developing spine is asymmetric. In most mdg embryos, vertebral fusion and defects of collagen structural arrangement in the AF were found on the ventral side, whereas the NP was displaced towards the dorsal side. Hayes *et al*. found matrix and cell behavioural differences between dorsal and ventral regions of the rat AF, as evidenced by the presence of versican in the dorsal region only, which could explain why these regions are affected differently by the lack of muscle contractions (Hayes *et al.*, 2001). Another explanation is that muscle contractions are required to “open up” the spine as it elongates along the craniocaudal axis. Indeed, it is known that muscle contractions are needed for elongation and reduction of the circumference of the embryo along the dorsoventral axis (Carvalho and Broday, 2020). Moreover, muscle contractions are necessary for the proper development of the neuromuscular junction (Felsenthal and Zelzer, 2017), which plays a key role in maintaining spinal alignment (Blecher *et al.*, 2017). The current findings were also consistent with observations on adolescent idiopathic scoliosis. Scoliotic patients often show abnormalities in their spinal and peripheral muscles, and these abnormalities are associated with a greater stress in the concave AF compared to the convex AF (reviewed in Wise *et al.*, 2020). Such asymmetric loading is the main factor that leads to morphological changes in the intervertebral disc, including a displacement of the NP toward the convexity of the curve. These studies on scoliotic patients further support the key role of muscle activity for the dorsoventral symmetry of the IVD.

The role of muscle forces for disc development appears to also differ across regions. Partial or complete vertebral fusion was found in the cervical region, associated with a disruption of the structural arrangement of the AF in most mdg embryos at TS27. In contrast, absence of muscle contraction led to wedging of the vertebral bodies in the thoracic region and did not affect vertebral body and disc morphology in the lumbar region. These results were in agreement with an earlier study on the effects of short-term immobilisation of chick embryos on spine development, which showed that abnormal spinal curvature and wedging of the vertebrae first appeared in the cervical region, and then progressively extended to the thoracic and lumbar regions as the spine grew (Levillain *et al.*, 2019). It is hypothesised that wedging of the vertebral bodies observed at TS27 would later lead to vertebral fusion and abnormal disc morphology as the spine grows. Indeed, Pai *et al*. found vertebral fusion in about 40 % of newborn mdg embryos in both cervical and thoracic regions (Pai, 1965). Given that vertebral and disc morphology appeared normal in the cervical region at TS24 but were disrupted at TS27, it is possible that the lumbar region would display vertebral and disc abnormalities later after birth, despite the absence of morphological defects at TS27. Alternatively, or potentially in addition, it is possible that the greater ranges of motion in the cervical region make them more dependent on physiological muscle loading.

The current study was not without limitations. The robustness of the results was limited by the small sample size, which was mainly due to the low breeding rates and therefore scarcity of mdg embryos. The effects of non-contractile muscles on disc development varied considerably between and within samples, with one mdg embryo being particularly severely affected. These differences between samples, observed mainly at TS27, may be due to the influence of passive movements (Nowlan *et al.*, 2012), which are likely more variable towards the end of prenatal development. The morphological characterisation was qualitative and not quantitative, due to the fact that the analyses were of 2D sections, which are dependent on the curvature on the spine and the section plane. Therefore, no reliable quantitative comparisons between the control and mdg groups were possible. Width and height measurements of the discs and vertebrae could be obtained using 3D imaging techniques, such as optical projection tomography (Rolfe *et al.*, 2017; Levillain *et al.*, 2019), but unfortunately it is not possible to combine this 3D imaging technique with immunofluorescence analyses. The immunofluorescence analyses were focused on the cervical region since it was the most affected (histologically) at TS27. It would be meaningful to study the lumbar region in more detail in later development, as it is the most affected by disc degeneration (reviewed in Kushchayev *et al.*, 2018), but this was not possible due to the neonatal lethality of the mdg mutation (Pai, 1965). Increasing the number of timepoints between TS24 and TS27 would provide a better understanding of the progression of effects and the link between altered mechanical properties and change in collagen structural arrangement, since absence of muscle contraction mostly affected IVD development between these two stages. Finally, further experiments need to be done to understand the mechanisms of how muscle forces affect IVD development. Indeed, the current study focused on the morphological and mechanical effects arising from the presence of muscle contractions, but other aspects of development such as biochemical signalling or collagen cross-linking may have also been affected. In particular, gene expression can co-vary significantly with the amount of mechanical stress (Rolfe *et al.*, 2014). Analysing the expression of key transcription factors and the chemical composition of the tissue would provide a more comprehensive understanding of the mechanisms involved in IVD development, including, but not limited to, the role of mechanical stimuli.

In conclusion, this study highlighted the role of muscle contractions for the development of disc morphology and mechanical properties. Notochord involution and vertebral segmentation took place normally in the absence of muscle contractions, but the intervertebral discs displayed several morphological defects, associated with changes in viscoelastic properties and vertebral fusion. Specifically, it was shown that muscle contractions were necessary for the structural arrangement of the inner AF between TS24 and TS27, and for the positioning of the NP along the dorsoventral axis. These findings provide new insights into the role of mechanical forces for disc development, highlight the importance of mechanical loading regimes in the development of the fibre reinforced annulus fibrosus, and suggest the presence and timing of mechanical stimuli are important factors for tissue engineering of replacement discs.

## Figures and Tables

**Table 1.** **Number of samples used for each type of analysis**.

Stage	Group	Number of samples			
		Histology	Immunofluorescence	Image analysis	Indentation
TS22	Control	3	3	-	-
	mdg	3	3	-	-
TS24	Control	3	3	3	3
	mdg	3	3	3	3
TS27	Control	3	3	3	3
	mdg	4	4	3	3

**Table 2.** Mean orientation of collagen II (relative to the dorsoventral axis) in control and mdg groups in each area at TS24 and TS27. Mean orientation ± standard deviation. Statistical analyses were not performed due to the low numbers of samples.

Area	TS24		TS27	
	Control (*n* = 3)	mdg (*n* = 3)	Control (*n* = 3)	mdg (*n* = 3)
Cranial-dorsal	93° ± 3.0°	95° ± 15°	70° ± 33°	75° ± 13°
Caudal-dorsal	90° ± 3.4°	107° ± 11°	109° ± 22°	85° ± 27°
Cranial-ventral	104° ± 32°	129° ± 19°	132° ± 8.9°	147° ± 7.8°
Caudal-ventral	87° ± 14°	7° ± 13°	53° ± 11°	150° ± 4.9°

**Table 3.** Instantaneous modulus and elastic fraction of vertebral bodies C5 and C6 and annulus fibrosus C5–C6 of control and mdg samples at TS24 and TS27. *F*-values and *p*-values of the statistical comparisons between age-matched control and mdg groups are indicated. E_ins_: instantaneous modulus. Mean ± standard deviation (3 samples per group with 6 to 10 indents per group).

Stage	Structure	Level	Group	E_ins_(kPa)	Elastic fraction
TS24	VB	C5	Control	29 ± 7.3	0.78 ± 0.046
			mdg	30 ± 15	0.74 ± 0.055
			*test statistics*	*F(1,4) = 0.019; p = 0.90*	*F(1,4) = 0.47; p = 0.53*
		C6	Control	29 ± 14	0.78 ± 0.024
			mdg	27 ± 9.5	0.77 ± 0.069
			*test statistics*	*F(1,4) = 0.10; p = 0.77*	*F(1,4) = 1.1; p = 0.34*
	AF	C5–C6	Control	24 ± 4.9	0.74 ± 0.042
			mdg	37 ± 21	0.67 ± 0.076
			*test statistics*	*F(1,4) = 0.85; p = 0.41*	*F(1,4) = 1.4; p = 0.31*
